# Foot kinematic and kinetic differences associated with eggbeater kick technique: a time-series analysis in water polo players

**DOI:** 10.3389/fspor.2026.1829900

**Published:** 2026-06-24

**Authors:** Eisuke Kawai, Daiki Koga, Yusaku Nakazono, Takaaki Tsunokawa, Keisuke Kobayashi Yamakawa, Hideki Takagi

**Affiliations:** 1 Institute of Health and Sport Sciences, University of Tsukuba, Tsukuba, Ibaraki, Japan; 2 School of Engineering, Institute of Science Tokyo, Meguro, Tokyo, Japan; 3 Advanced Research Initiative for Human High Performance (ARIHHP), University of Tsukuba, Tsukuba, Ibaraki, Japan

**Keywords:** aquatic sports, motion analysis, pressure distribution analysis, statistical parametric mapping, treading water

## Abstract

Eggbeater kicking is essential for maintaining body position and supporting technical actions in water polo, yet the time-specific biomechanical characteristics associated with higher performance remain unclear. This study aimed to identify the characteristics of water polo players with higher eggbeater kicking performance by comparing foot kinematics and kinetics using time-series analysis. Twenty-two male university players performed continuous eggbeater kicking while underwater motion capture and pressure sensors recorded foot movements and pressure distributions. Resultant fluid forces and vertical propulsive forces were estimated using pressure distribution analysis. Participants were allocated into upper and lower groups using a pre-set threshold for normalised propulsive force derived from an independent dataset. Statistical parametric mapping (one-tailed SPM{t}, *α* = 0.05) was applied to detect time-specific between-group differences. Time-specific differences in dynamic pressure and foot angles were identified mainly during the out-kick phase, whereas no significant differences were observed in foot velocity or acceleration. These findings suggest that the timing of foot orientation adjustments, rather than movement velocity alone, may be an important characteristic associated with propulsion-related performance during eggbeater kicking. The identified critical phases may inform targeted technical training and guide future biomechanical investigations of unsteady propulsion mechanisms.

## 1 Introduction

Eggbeater kick is a water-treading technique in which athletes continuously alternate circular movements of their lower limbs to generate upward propulsive force and maintain a high body position in the water. This technique is often used in underwater activities such as water polo, artistic swimming and lifesaving. The generation of propulsive force by the eggbeater kick enables a variety of actions by water polo players, such as passing, shooting and blocking, to maximise their performance (1–5). Therefore, performing an effective eggbeater kick is important for all water polo players.

Due to its important role in propulsion generation, the foot during the eggbeater kick has been analysed from both kinematics and kinetics. It has been observed that the feet of elite water polo players and artistic swimmers during the eggbeater kick maintain high speeds throughout the cycle with a movement trajectory that is more mediolateral and less vertical (6, 7). Negative attack angles (i.e., movement toward the dorsal side of the foot) should also be minimised to avoid generating downward forces that impede propulsion (8, 9). Maximum attack angle (usually observed during the out-kick phase, i.e., the phase in which the foot moves outwards from the body and diagonally downward) is also recognised as an important parameter to identify the eggbeater kick technique (7, 10). Furthermore, recent studies (10, 11) have shown that the propulsive force during the eggbeater kick is increased by the pressure difference between the plantar and dorsal side of the foot, which is mainly influenced by the decrease in pressure on the dorsal side of the foot. Such pressure fluctuations are likely influenced by unsteady flow around the foot. Although vortex visualisation during eggbeater kicking has not yet been conducted, similar pressure-driven mechanisms have been observed in hand sculling (12). Collectively, these studies established the biomechanical basis for understanding eggbeater kicking, including pressure-based force estimation and candidate kinematic and kinetic variables related to propulsion performance. However, they did not statistically test whether continuous time-series waveforms differ between players with different levels of eggbeater kick performance.

Accordingly, the remaining question is not simply which variables are relevant, but when within the kick cycle performance-related differences become evident across players of different performance levels, because previous studies have not statistically compared continuous eggbeater kick waveforms between players classified according to objectively quantified propulsion performance (6, 7, 10). Thus, it remains unclear which foot movements distinguish more effective kickers from less effective ones. Moreover, identifying the specific time windows in the kick cycle where differences emerge is important for gaining deeper and more detailed insights that can inform coaching practices (e.g., designing targeted technical drills) (13). This is particularly important for eggbeater kicking because propulsion is generated continuously through cyclical and rapidly changing foot movements, so brief technique-related deviations may be obscured when only discrete values are considered. Under unsteady flow conditions, pressure distribution and fluid force can change rapidly over short durations (10, 11, 14), yet the precise movement features responsible for these fluctuations remain unclear. Although movements may appear similar at first glance, performance differences may result from subtle, instantaneous variations that are not captured by discrete phase-averaged values.

To address these gaps, the present study applies time-series statistical parametric mapping (SPM) to identify the specific periods of the kick cycle during which superior technique becomes evident, an issue that cannot be resolved using discrete phase-averaged variables alone. Therefore, the aim of this study was to identify the characteristics of water polo players with superior eggbeater kick technique by comparing foot kinematics and kinetics time-series between groups. We hypothesised that the higher-performance group would exhibit more favourable kinetic values (i.e., greater plantar pressure and propulsion-related forces, and lower dorsal pressure) together with more effective foot orientation during the out-kick phase, particularly when the foot moves diagonally downward.

## 2 Materials and methods

### 2.1 Sample size estimation and justification

Because no established *a priori* power analysis framework is currently available for the present time-series SPM design, sample size was estimated as an approximate planning reference using a one-tailed independent two-sample t-test (*α* = 0.05, *β* = 0.20). The one-tailed design was selected *a priori* because previous studies on eggbeater kicking suggested directional relationships between propulsion-related variables and movement characteristics during the out-kick phase (7, 8, 10). Specifically, greater propulsion has been associated with more favourable foot orientation and pressure-related characteristics, rather than with opposite-direction effects. Accordingly, the present study tested the hypothesised direction of difference between higher- and lower-performance players. The expected effect size (Cohen's *d* = 1.18) was adopted from competitive front-crawl swimming literature reporting large within-subject effects in propulsion-related variables (e.g., 14, 15). Although this reference did not perfectly match the present between-group design and may not fully represent the biomechanical context of eggbeater kicking, it was used as the closest available approximation in the absence of comparable effect size estimates for this movement. Therefore, the sample size estimation should be interpreted as a pragmatic guide rather than as a design-specific SPM power analysis, and the statistical sensitivity of the present study should be considered exploratory in nature. Based on these parameters, G*Power (version 3.1.9.7; Heinrich Heine University, Germany) indicated that at least 10 participants per group were required.

### 2.2 Participants

Participants were twenty-two national-level male university water polo players (age: 21.0 ± 1.4 years, height: 1.77 ± 0.07 m, body mass: 80.0 ± 10.9 kg, competitive experience: 9.1 ± 3.0 years, dominant foot: right). At the time of this study, the participants had six sessions of water polo training per week. The present participants constituted a newly recruited cohort and were not included in previously published studies on eggbeater kick biomechanics (10, 11, 16). Each participant received an oral explanation of the potential risks and benefits of the study and gave written informed consent to participate. The study design and risks were reviewed and approved by the institutional Research Ethics Committee of the authors’ university (Approval No. 29-134).

### 2.3 Experimental setup

**Figure 1:**
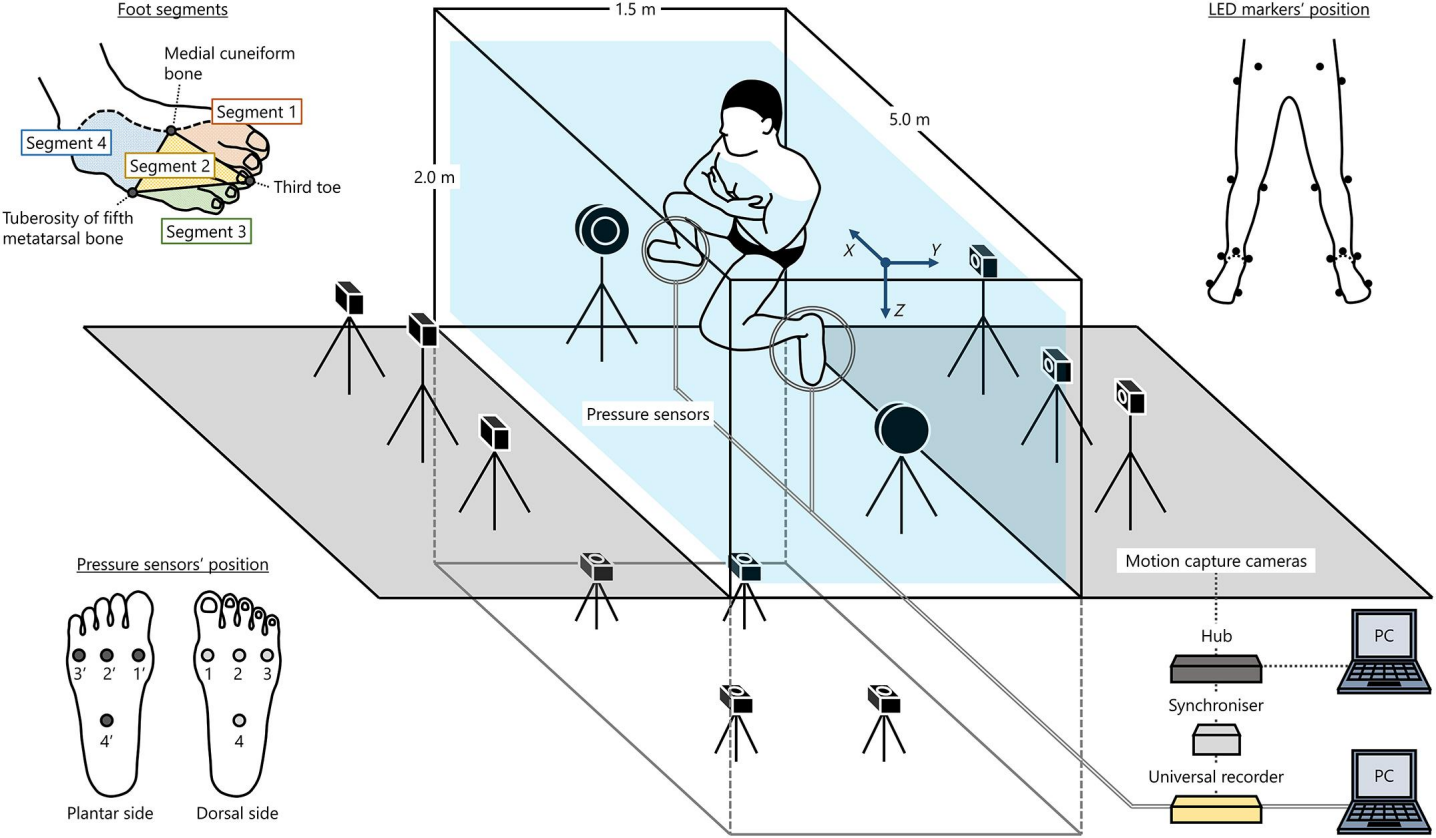
Schematic representation of the experiment. The testing was performed in an experimental aquatic flume. The participants’ eggbeater kicking motions were recorded by a motion capture system composed of twelve cameras. The pressure distributions around the feet were measured by sixteen waterproof pressure sensors attached to the dorsal and plantar surfaces of the participants’ both feet.

Testing was performed in an experimental aquatic flume with underwater glass windows based on Kawai at al (10). (Figure 1). The participants performed maximal effort eggbeater kicks with no arm sculls, aiming to maintain the highest possible body position. The participants were instructed to cross their arms in front of the chest and hold their breath during the trial. Their eggbeater kick motions were recorded using a motion capture system composed of twelve cameras (OptiTrack, NaturalPoint, Inc., USA, sampling frequency 100 Hz). Two cameras were positioned in the flume, and ten cameras were located outside the flume with the cameras viewing the testing space through the windows (three front cameras, three back cameras, and four bottom cameras). Anatomical landmarks (left-right greater trochanters, superior anterior iliac spines, knee joints, ankle joints, first and fifth toes and heels; total eighteen points) were marked by wireless light-emitting diode markers (Kirameki, Nobby Tech. Ltd., Japan). To measure the pressure distribution around the feet, sixteen waterproof pressure sensors (PS-05KC, Kyowa Electronic Instruments Co. Ltd., Japan) were attached to the right and left foot (four each on the dorsal and plantar surfaces). The data measured by the pressure sensors were recorded on a laptop computer with a sampling frequency of 100 Hz via a universal recorder (EDX-100A, Kyowa Electronic Instruments Co. Ltd., Japan). The measurements were taken for 5 s in the middle of each trial lasting approximately 12 s to ensure steady-state performance. The motion and pressure data were synchronised by a dedicated synchroniser (eSync, NaturalPoint, Inc., USA).

### 2.4 Definition of coordinate systems

The measurement area was calibrated using a dynamic calibration method, resulting in a standard calibration error of less than 0.0003 m. A global right-handed coordinate system (*X*-*Y*-*Z*) was defined using a dedicated base plate (Figure 1). In this system, the *X*-axis represented the left-right horizontal direction, the *Y*-axis represented the front-back horizontal direction relative to the participant's facing direction, and the *Z*-axis represented the vertical direction (positive downward).

**Figure 2:**
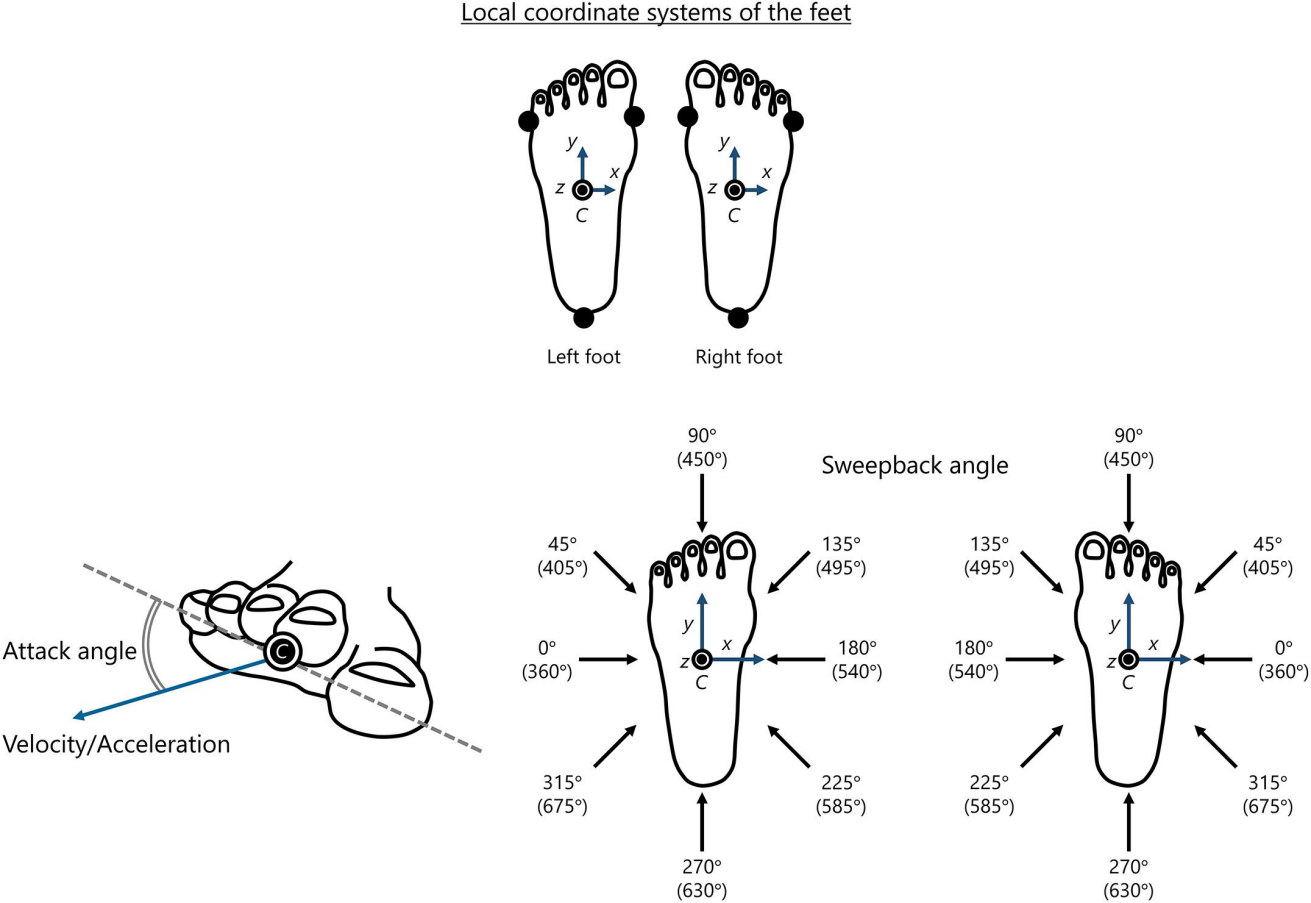
Local right-handed coordinate systems of the feet and kinematic foot parameters.

To describe foot orientation and motion in a foot-specific manner, local right-handed coordinate systems (*x*-*y*-*z*) were defined for the right and left feet (Figure 2). The origin of each local coordinate system was set at the centre (*C*) of the plane formed by the first toe, fifth toe, and heel. The *y*-axis was defined by the line connecting the heel to *C*, with the positive direction toward the toes. The *x*-axis was defined within the same plane and perpendicular to the *y*-axis; for the right foot, the positive direction was toward the fifth toe, whereas for the left foot, it was toward the first toe. The *z*-axis was defined as perpendicular to both the *x*- and *y*-axes.

### 2.5 Kinematic foot parameters and motion structure of eggbeater kick

The 3D coordinates of the anatomical landmarks recorded by the motion capture system were filtered using a low-pass Butterworth digital filter with a cut-off frequency of 6 Hz (8, 9). The kinematic variables analysed in this study were the attack angle, the sweepback angle, and the resultant velocity and acceleration of *C* (Figure 2).

The attack angle describes how the foot is tilted relative to its direction of motion. It was calculated as the angle between the velocity vector of *C* and the plane of the foot (8, 10). The sweepback angle describes the direction of foot motion within the foot plane and therefore reflects the direction of relative water inflow to the foot. It was calculated as the angle between the projection of the velocity vector of *C* onto the foot plane and the *x*-axis of the foot local coordinate system (10, 17). For the right foot, the sweepback angle was defined as positive in the counter-clockwise direction, with 0° (360°) representing complete overlap between the projected velocity vector and the *x*-axis in the foot plane. The left sweepback angle was defined as the mirror image of the right sweepback angle.

One cycle of the eggbeater kick was defined as the period between two sequential maximally flexed positions of the right knee. Within one cycle, the motion was divided into two phases: the out-kick, from maximal knee flexion to maximal knee extension, and the in-kick, from maximal knee extension to maximal knee flexion (6, 10). Because the right and left legs move out of phase during eggbeater kicking, the right and left legs began the cycle from the out-kick and in-kick phases, respectively.

### 2.6 Estimation of fluid force (resultant force and propulsive force)

Fluid forces were estimated using the pressure distribution analysis (PDA) method described in previous studies (10, 11, 16, 18). This method estimates the fluid forces acting on body segments from the pressure difference between the plantar and dorsal surfaces and is suited to analysing unsteady propulsion in water.

For the present analysis, the foot was divided into four segments (segments 1–4: around the first toe, third toe, fifth toe, and heel, respectively), and a pair of pressure sensors was attached to the dorsal and plantar side of each segment (Figure 1). The measured pressure data were filtered using a low-pass Butterworth digital filter with a cut-off frequency of 8.3 Hz, determined by residual analysis. Because the recorded pressure included both dynamic pressure and static pressure due to sensor depth, the static pressure component was removed. To do this, the depth of each pressure sensor was estimated from the foot coordinates (first toe, fifth toe, and heel), and the corresponding static pressure was subtracted so that only dynamic pressure remained.

The entire dynamic pressures acting on the dorsal and plantar sides of the foot (*P_dorsal_* [N/m^2^] and *P_plantar_* [N/m^2^]) were then calculated using Equations 1 and 2, respectively, following Koga et al. (14):

$$P_{dorsal}=\frac{\sum_{i=1}^{4}\;P_{dorsal\_i}\times A_{i}}{A}$$ (1)

$$P_{\;plantar}=\frac{\sum_{i=1}^{4}\;P_{\;plantar\_i}\times A_{i}}{A}.$$ (2)

where *P_dorsal_i_* (N/m^2^) indicates the dynamic pressure on the *i*th segment of the dorsal side of the foot (*i* = 1–4); *P_plantar_i_* (N/m^2^), the dynamic pressure on the *i*th segment of the plantar side; *A_i_* (m^2^), the projected area of the *i*th segment; and *A* (m^2^), the entire projected area of the foot.

The fluid force acting on each segment was estimated using Equation 3:

$$F_{segment\_i}=A_{i}\times P_{differ\_i\;(i\;=\;1-4)}$$ (3)

where *F_segment_i_* (N) indicates the fluid force acting on the *i*th segment of the foot, and *P_differ_i_* (N/m^2^) is the plantar–dorsal pressure difference on the *i*th segment (*P_plantar_i_*−*Cos θ_i_ P_dorsal_i_*). Because the plantar and dorsal surfaces are not parallel, the pressure difference was adjusted using the sagittal plane angle (*θ_i_*) between the paired pressure sensors attached to the plantar and dorsal surfaces, measured in the standing position.

The resultant fluid force acting on the entire foot (*F*_*foot*_ [N]) was then estimated using Equation 4:

$$F_{\;foot}=\overset{4}{\underset{i=1}{\sum }}\;F_{segment\_i}$$ (4)

Because pressure acts perpendicular to a surface, and because the present calculation was based on the plantar surface of the foot, *F_foot_* was considered to act perpendicular to the plantar side of the foot and parallel to the normal vector of the foot plane, calculated as the cross product of the heel–fifth toe and heel–first toe vectors. The vertical component of *F_foot_* (*F_z_* [N]) was obtained by multiplying *F_foot_* by the *Z* component of the unit normal vector of the foot plane. This vertical fluid force (*F_z_*) was regarded as the propulsive force generated during eggbeater kicking and was defined as positive when acting upward.

### 2.7 Statistical analysis

Based on the results of a previous study (10), which reported that the left-right average propulsive force normalised by body mass had the largest influence on clustering performance levels in eggbeater kicking, participants were assigned to two groups using an *a priori*, fixed threshold (median = 0.913 N/kg) derived from that independent dataset (see Table 1). That previous dataset was used solely to define the threshold, and no participants from that dataset were included in the present study. The threshold was determined independently of the present sample and was not derived *post hoc* from the current data. Mean propulsive force was used solely for group allocation; all statistical comparisons were performed on the time-series kinetic and kinematic variables obtained in the present measurements. The purpose of this grouping procedure was not to reduce propulsion to a binary outcome, but to enable a direct comparison of time-series waveform patterns between players with relatively higher and lower propulsion-related performance. This between-group contrast was considered appropriate for the present study because the primary aim was to identify when within the kick cycle technique-related differences emerge between performance levels, rather than to examine propulsion as a continuous variable. Although treating propulsion as a continuous variable may also provide useful information, such an approach was beyond the scope of the present study. A similar grouping-based time-series approach has also been adopted in previous research on underwater undulatory swimming (19), in which swimmers were grouped according to average swimming velocity and time-series variables, including swimming velocity and joint angles, were compared over one kick cycle.

**Table 1:** Descriptive statistics for the left-right average propulsive force normalised by body mass (N/kg) used for group classification.

Group	Threshold	Mean	SD	Median	Max-Min
Upper (*n* = 11)	> 0.913	1.05	0.11	1.03	0.36
Lower (*n* = 11)	≤ 0.913	0.73	0.09	0.73	0.28

The pre-set threshold was adopted from the median value obtained in a previous independent dataset (10). No between-group inference was conducted.

SPM based on t-statistics (20) was used to compare kinetic and kinematic foot time-series data between groups. While SPM is an established approach in swimming and aquatic biomechanics (13, 19, 21–24), its application in eggbeater kicking allowed identification of transient features within the cycle that may not be captured by traditional discrete analyses. For each variable, the time-series data for one eggbeater kick cycle, defined as the period between two sequential maximally flexed positions of the right knee, were time-normalised to 101 data points (0%–100%) in MATLAB (version R2021a; The Mathworks, Inc., USA) prior to the SPM analysis. The SPM analyses were performed using the spm1D package (version M.0.4.10, https://spm1d.org/) in MATLAB with one-tailed inference (*α* = 0.05), consistent with the *a priori* directional hypothesis that higher-performance players would exhibit more favourable propulsion-related kinetic and kinematic characteristics during the out-kick phase.

## 3 Results

**Figure 3:**
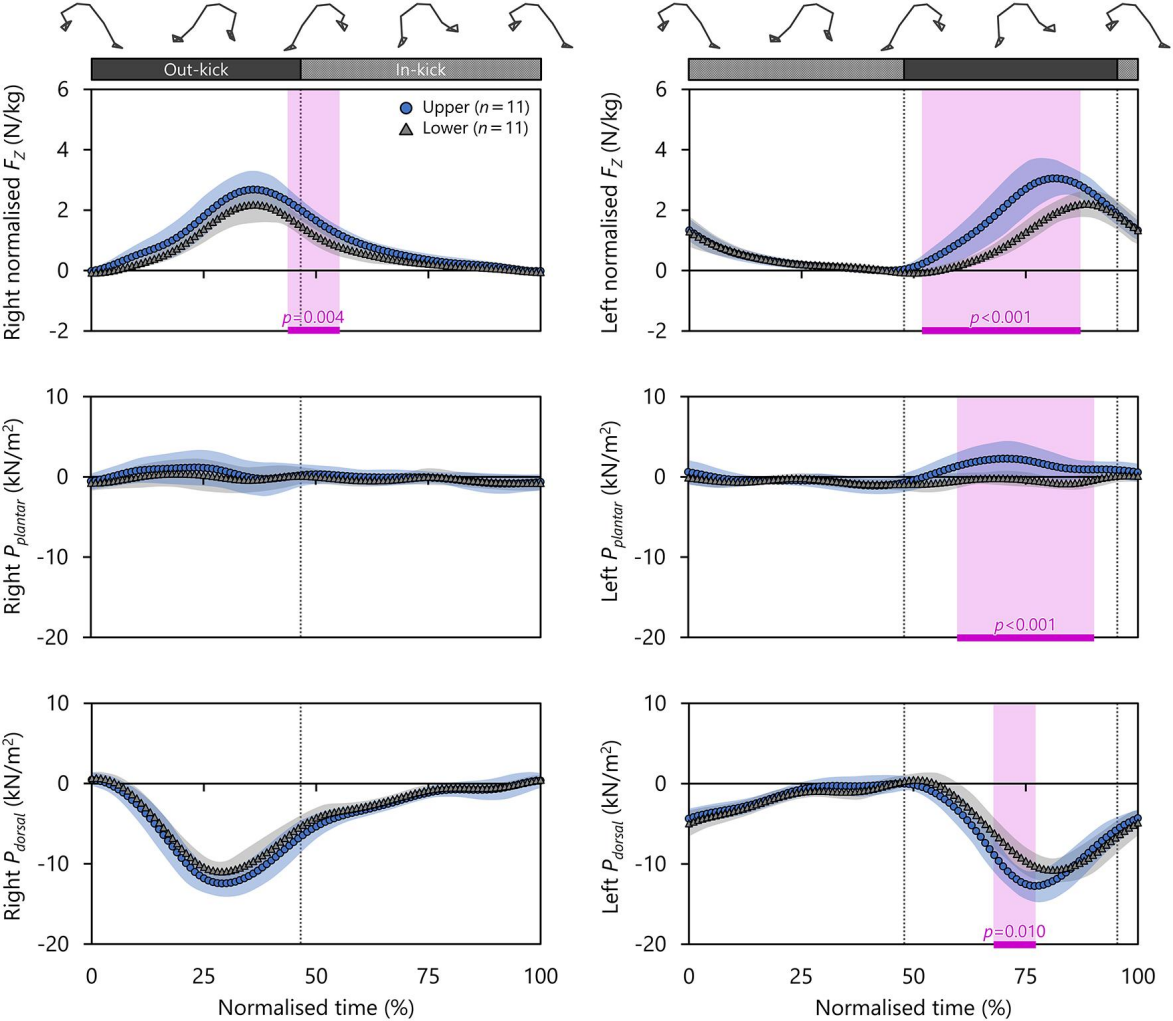
Fluctuations in normalised propulsive force (*F_z_*) and dynamic pressures (*p_plantar_* and *p_dorsal_*) during one eggbeater kick cycle for the upper and lower groups (each averaged over *n* = 11). Stick figures illustrate the eggbeater kicking motion viewed from the frontal plane. Dark- and light-grey bars indicate the out-kick and in-kick phases, respectively. For the right foot, the cycle begins with the out-kick phase, whereas for the left foot, the cycle begins with the in-kick phase; dotted vertical lines indicate the phase boundaries within the normalised cycle. Pink-shaded regions represent time windows in which significant between-group differences were observed.

**Figure 4:**
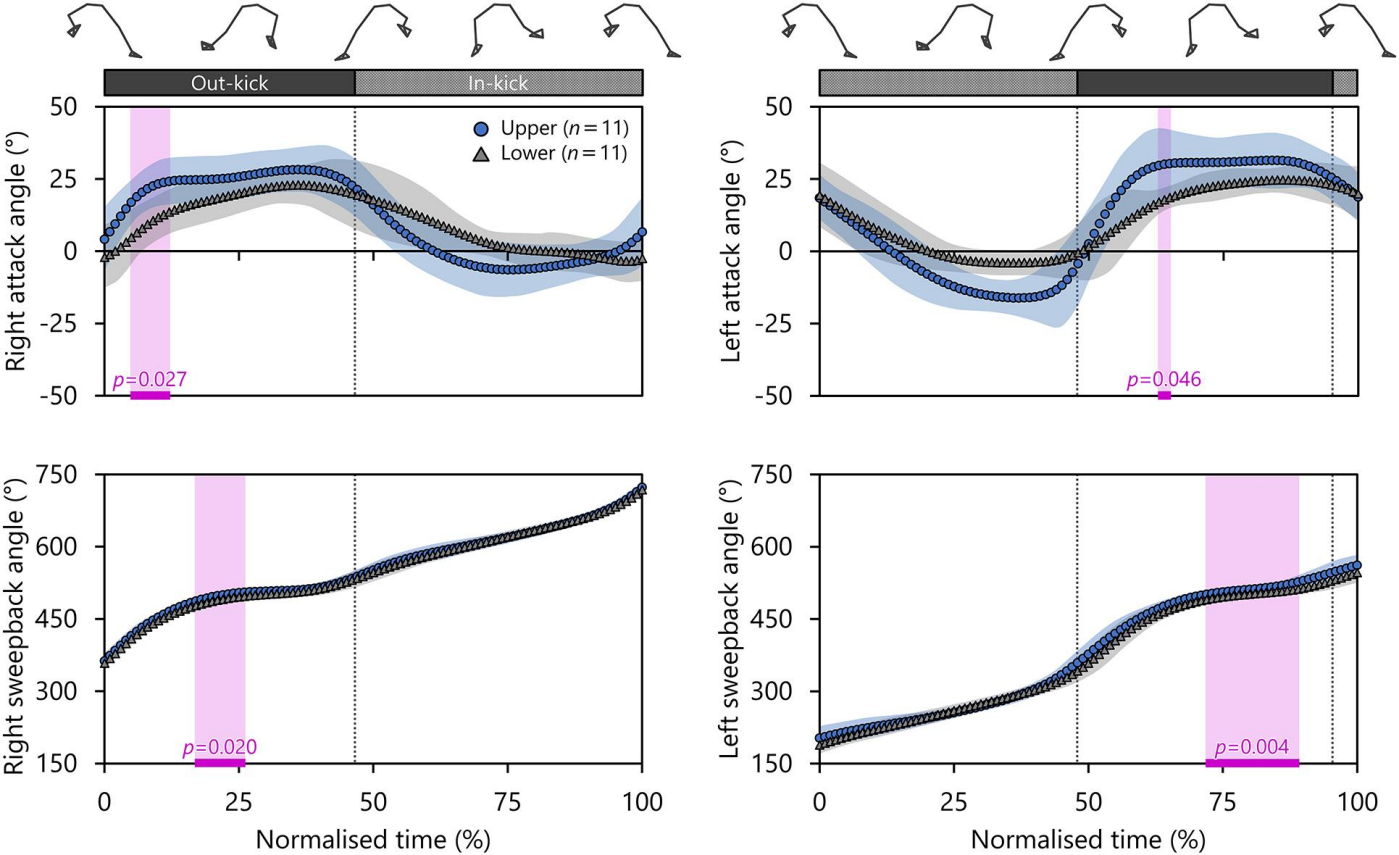
Fluctuations in attack and sweepback angles during one eggbeater kick cycle for the upper and lower groups (each averaged over *n* = 11). Stick figures illustrate the eggbeater kicking motion viewed from the frontal plane. Dark- and light-grey bars indicate the out-kick and in-kick phases, respectively. For the right foot, the cycle begins with the out-kick phase, whereas for the left foot, the cycle begins with the in-kick phase; dotted vertical lines indicate the phase boundaries within the normalised cycle. Pink-shaded regions represent time windows in which significant between-group differences were observed.

**Figure 5:**
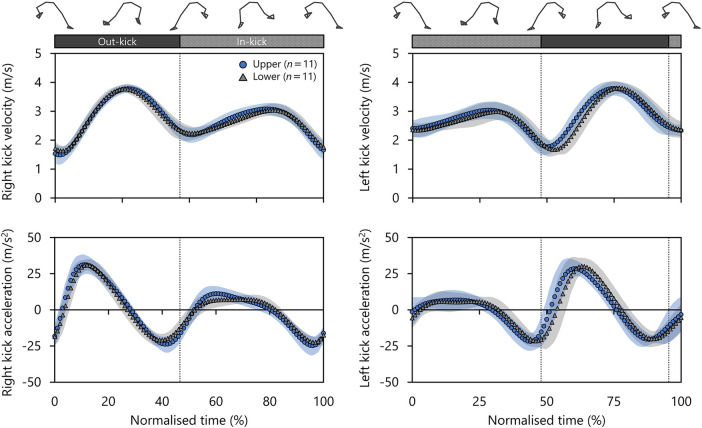
Fluctuations in kick velocity and acceleration during one eggbeater kick cycle for the upper and lower groups (each averaged over *n* = 11). Stick figures illustrate the eggbeater kicking motion viewed from the frontal plane. Dark- and light-grey bars indicate the out-kick and in-kick phases, respectively. For the right foot, the cycle begins with the out-kick phase, whereas for the left foot, the cycle begins with the in-kick phase; dotted vertical lines indicate the phase boundaries within the normalised cycle. No significant between-group differences were detected.

Quantitative summaries of all significant time windows identified by the one-tailed SPM{t} analyses, including effect sizes and confidence intervals, are provided in Table 2. Time-series comparisons of foot kinetics and kinematics between the groups are shown in Figures 3–5. Significant between-group differences in the normalised propulsive force (*F_z_*) of the right foot were observed around the boundary between the two kick phases (44%–55% of the cycle), whereas those of the left foot were found during a large part of the out-kick phase (52%–87%). Significant between-group differences in the dynamic pressures (*P_plantar_* and *P_dorsal_*) of the left foot were observed during the out-kick phase (60%–90% and 68%–77%, respectively), while no significant differences were detected in the right foot. Significant between-group differences in attack angle were observed during the early portion of the out-kick phase for both feet (right: 5%–12%; left: 63%–65%), and significant differences in sweepback angle were found during the mid-to-late portion of the out-kick phase (right: 17%–26%; left: 72%–89%). No significant between-group differences in kick velocity or acceleration were identified at any point during the kick cycle. Overall, the significant time windows were more extensive in the left foot than in the right foot.

**Table 2:** Quantitative summary of significant time windows identified by one-tailed SPM{t} analyses.

Variable	Side	Significant time window	*p*	*t*	Mean difference	SD difference	95% CI	Cohen's *d*
Normalised *F_z_* (N/kg)	Right	44–55%	0.004	3.056	0.48	0.34	[0.24, 0.72]	1.67
	Left	52–87%	< 0.001	3.049	0.97	0.62	[0.59, 1.34]	2.16
*P_plantar_* (kN/m^2^)	Right	n.s.	–	–	–	–	–	–
	Left	60–90%	< 0.001	2.927	2.08	1.94	[1.04, 3.11]	1.67
*P_dorsal_* (kN/m^2^)	Right	n.s.	–	–	–	–	–	–
	Left	68–77%	0.010	−3.011	−2.95	2.88	[−4.39, −1.51]	−1.71
Attack angle (°)	Right	5–12%	0.027	2.944	11.80	13.02	[5.03, 18.56]	1.46
	Left	63–65%	0.046	2.978	12.17	13.96	[4.21, 20.13]	1.28
Sweepback angle (°)	Right	17–26%	0.020	2.889	8.79	10.04	[3.66, 13.92]	1.43
	Left	72–89%	0.004	2.888	10.68	11.37	[4.24, 17.13]	1.39
Kick velocity (m/s)	Right	n.s.	–	–	–	–	–	–
	Left	n.s.	–	–	–	–	–	–
Kick acceleration (m/s^2^)	Right	n.s.	–	–	–	–	–	–
	Left	n.s.	–	–	–	–	–	–

Positive values indicate upper > lower, and negative values indicate upper < lower. Mean difference (upper−lower) and Cohen's *d* share the same sign.

## 4 Discussion

The present study identified time-specific differences in foot kinetics and kinematics between water polo players with higher and lower eggbeater kicking performance, mainly during the out-kick phase. In particular, significant between-group differences were observed in attack and sweepback angles (Figure 4), as well as in dynamic pressure and normalised propulsive force (Figure 3), whereas no significant differences were found in kick velocity or acceleration (Figure 5). These differences were more extensive in the left foot than in the right foot, especially for propulsive force and dynamic pressure. Taken together, these results suggest that propulsion-related performance during eggbeater kicking may be differentiated more by how the foot is oriented and how pressure is distributed over the foot surface during specific periods of the cycle than by movement velocity alone. Because the groups were defined according to overall propulsive performance, the greater propulsion observed in the upper group should be interpreted as a defining group characteristic rather than as an independent outcome.

Although velocity and acceleration are important contributors to propulsion (7, 25, 26), no between-group differences were detected in these variables. One possible interpretation is that both groups had already attained a comparable baseline level of movement velocity, and that the key difference lay in how effectively that motion was oriented relative to the surrounding water. A similar interpretation has been suggested in front-crawl swimming, where increasing hand velocity at an excessively high stroke frequency did not increase propulsive force, because the attack angle decreased at the same time (27). This suggests that movement velocity alone may be insufficient to enhance propulsion if the orientation of the propulsive surface is not effectively controlled. In this context, the present findings suggest that the fine control of foot orientation may play a more critical role than movement velocity itself in differentiating propulsion-related performance. Importantly, while earlier work reported associations between foot angles and propulsion effectiveness during the eggbeater kick (e.g., 6, 7, 10), it did not determine when such differences emerged within the kick cycle. The present results extend this understanding by identifying the specific periods during which technique-related differences were most clearly expressed. Although these associations align with theoretical expectations and findings in other aquatic propulsion tasks (e.g., 27, 28), they should not be interpreted causally. The present design does not allow determination of whether more favourable foot angles contributed to greater propulsion, or whether players with greater propulsion tended to exhibit more favourable foot angles.

The between-group difference in propulsive force, particularly on the non-dominant side, was most pronounced during the part of the out-kick phase when the foot moved diagonally downward (Figure 3). During this period, the upper group exhibited not only a more rapid increase in attack angle but also a more medially directed sweepback angle (toward the first toe side) (Figures 2, 4). These localised kinematic differences preceded or accompanied the kinetic differences observed later in the cycle, including differences in dynamic pressure and propulsive force. Although surface pressure and kinematic data alone cannot determine the underlying fluid mechanics, this temporal sequence suggests that time-specific foot orientation adjustments may be associated with a more favourable pressure distribution on the foot surface and, in turn, enhanced propulsive output during the out-kick. This time-specific evidence helps identify critical phases for technique optimisation and provides direction for future mechanistic investigations [e.g., particle image velocimetry (PIV) or computational modelling]. From a practical perspective, these findings suggest that technique instruction may be most effective when focused on foot orientation control during the out-kick, particularly around the period when the foot moves diagonally downward. Indeed, a hand-sculling study has shown that medially directed motion (i.e., in-scull) combined with appropriate attack angle control can generate leading-edge vortices that increase palm–dorsal pressure differentials and enhance propulsion (12).

This study has several limitations and considerations. First, the experimental setup constrained arm motion and breathing to isolate the propulsive contribution of the eggbeater kick itself; therefore, the present findings reflect the mechanical potential of lower-limb technique rather than the full ecological performance observed in gameplay, where stability, endurance, and coordination with arm actions are also required. Second, although the underwater motion capture system was calibrated with minimal error (< 0.0003 m) and marker visibility was well maintained under the present experimental conditions, soft-tissue artefacts and occasional occlusion remain inherent limitations of underwater optical tracking. Third, while the PDA method is advantageous for analysing foot-generated propulsion under unsteady flow conditions, it cannot estimate the total lower-limb propulsive output of the eggbeater kick. In addition, although some significant time windows showed large effect sizes, these values should be interpreted with caution because effect size estimates may be unstable in relatively small samples. Accordingly, the present study may have been underpowered to detect smaller but potentially meaningful biomechanical differences. The more extensive between-group differences observed in the non-dominant (left) foot may reflect task-specific functional asymmetries in eggbeater kicking (9, 29), but this interpretation remains tentative because no direct measures of neuromuscular control or coordination asymmetry were collected. Finally, different results may be obtained in players of different sex or proficiency levels, such as novices.

## 5 Conclusion

The present study identified time-specific kinetic and kinematic differences between water polo players with higher and lower eggbeater kicking performance. Although the greater propulsive force observed in the upper group reflects the grouping criterion itself, differences in dynamic pressure and foot angles were identified mainly during the out-kick phase. In contrast, no between-group differences were found in foot velocity or acceleration. These findings suggest that the timing and control of foot orientation, rather than movement velocity alone, may be an important characteristic associated with propulsion-related performance during eggbeater kicking. The identified time windows may help inform technique-focused training and future biomechanical investigations.

## Data Availability

The original contributions presented in the study are included in the article/supplementary material, further inquiries can be directed to the corresponding author.
